# Association between DNMT3A Mutations and Prognosis of Adults with *De Novo* Acute Myeloid Leukemia: A Systematic Review and Meta-Analysis

**DOI:** 10.1371/journal.pone.0093353

**Published:** 2014-06-17

**Authors:** Ruxiu Tie, Tiansong Zhang, Huarui Fu, Limengmeng Wang, Yebo Wang, Ying He, Binsheng Wang, Ni Zhu, Shan Fu, Xiaoyu Lai, Jimin Shi, He Huang

**Affiliations:** 1 Bone Marrow Transplantation Center, The First Affiliated Hospital, Zhejiang University School of Medicine, Zhejiang, China; 2 Department of TCM, Jing'an District Central Hospital, Shanghai, China; B.C. Cancer Agency, Canada

## Abstract

**Background:**

DNA methyltransferase 3A (DNMT3A) mutations were considered to be independently associated with unfavorable prognosis in adults with de novo acute myeloid leukemia (AML), however, there are still debates on this topic. Here, we aim to further investigate the association between DNMT3A mutations and prognosis of patients with AML.

**Methods:**

Eligible studies were identified from several data bases including PubMed, Embase, Web of Science, ClinicalTrials and the Cochrane Library (up to June 2013). The primary endpoint was overall survival (OS), while relapse-free survival (RFS) and event-free survival (EFS) were chosen as secondary endpoints. If possible, we would pool estimate effects (hazard ratio [HR] with 95% confidence interval[CI]) of outcomes in random and fixed effects models respectively.

**Results:**

That twelve cohort studies with 6377 patients exploring the potential significance of DNMT3A mutations on prognosis were included. Patients with DNMT3A mutations had slightly shorter OS (HR = 1.60; 95% CI, 1.31–1.95; P<0.001), as compared to wild-type carriers. Among the patients younger than 60 years of age, DNMT3A mutations predicted a worse OS (HR = 1.84; 95% CI, 1.36–2.50; P<0.001). In addition, mutant DNMT3A predicted inferior OS (HR = 2.30; 95% CI, 1.78–2.97; P = 0.862) in patients with unfavorable genotype abnormalities. Similar results were also found in some other subgroups. However, no significant prognostic value was found on OS (HR = 1.40; 95% CI, 0.98–1.99; P = 0.798) in the favorable genotype subgroup. Similar results were found on RFS and EFS under different conditions.

**Conclusions:**

DNMT3A mutations have slightly but significantly poor prognostic impact on OS, RFS and EFS of adults with de novo AML in total population and some specific subgroups.

## Introduction

Acute myeloid leukemia (AML) are a group of heterogeneous diseases with respect to biological and clinical outcomes, which have been considered to be related to cytogenetic and gene lesions in hematopoietic stem or progenitor cells [1], [2], [3]. It is extremely important for patients with de novo AML to assess risk status based on cytogenetic and molecular abnormalities. Cytogenetic abnormalities have been utilized widely to evaluate the risk status of patients [3], [4], [5], [6]. However, only several molecular lesions were applied to risk stratification in clinical practice, such as FMS-like tyrosine kinase 3 (FLT3) [7], [8], Nucleophosmin1 (NPM1) [9], [10], CCAAT/enhancer-binding protein alpha (CEBPA) [11], [12]. Whereas DNMT3A [13], [14], [15], Isocitrate dehydrogenase1 (IDH1), IDH2 [16], [17] and TET2 [18], [19] have not been fully assessed. DNMT3A plays a role in de novo methylation of specific CpG islands in DNA, and DNMT3A mutations are implicated in the pathogenesis and prognosis of patients with AML [20]. Recurrent abnormities of the gene include missense, frameshift, nonsense, splice-site mutations and part deletion [13] as well. The most common type of missense mutations is predicted to affect the amino acid at R882, which accounts for more than half the population with DNMT3A mutations [13], [21], [22]. These mutations are highly enriched in the group of adults with an intermediate-risk cytogenetic profile or with cytogenetic normal AML (CN-AML) [13], [22], [23], [24], but they are absent or scarce in patients with a favorable-risk cytogenetic profile [21], [23], [24]. DNMT3A mutations having negative impact on OS of patients with AML were reported in several studies but not in the others [20], [22], [25], [26]. Similar discrepancies were observed in terms of RFS and EFS. Thus it is necessary to perform a systematic review and meta-analysis to further clarify the prognostic values of mutant DNMT3A in patients with de novo AML.

## Materials and Methods

### 1 Literature search and search strategy

Literature search was conducted in several electronic databases including PubMed, Embase, Web of Science, ClinicalTrials and the Cochrane Library (up to June 26, 2013). We used the following search terms (medical subject headings [MeSH] or key words) limited in titles and abstracts: “acute myeloid leukemia”, “acute myeloblastic leukemia”, “acute myelocytic leukemia”, “acute granulocytic leukemia”, “Leukemia, Myeloid, Acute”, “AML”, and “DNA methyltransferase 3A”, “DNA (cytosine-5)-methyltransferase 3A”, “DNMT3A”. No other search terms were added in order to obtain more related studies.

### 2 Selection criteria

If meeting the following selection criteria, studies should be included: (1) studies published in English; (2) Cohort studies or meeting abstracts; (3) studies focusing on prognostic impact of DNMT3A mutations in adults with de novo AML; (4) data from time-to-event analysis in every study; (5) the latest version of multiple publications or overlap of some context from the same cohort should be selected, the older version should be used to clarify methodology or characteristics of patients.

Two investigators (R.-x.T. and T.-s.Z.) were asked to identify the eligible studies independently. Endnote ×6 were used for the management of the articles and for removing most of the duplicates. Unrelated articles and remaining duplicates were excluded by reading the abstracts carefully. The remaining full-text articles were retrieved and reviewed carefully to identify the eligible studies. Any divergency was resolved by asking a third author's opinion (H.-r.F.). The selection process was documented in a flow chart recommended in the PRISMA statement.

### 3 Risk of bias from included studies

The quality of eligible studies was assessed independently by two reviewers (R.-x.T. and T.-s.Z.) using the Newcastle-Ottawa-Scale (NOS) [27] for cohort studies. The NOS assigns a maximum of 4 points for selection, 2 points for comparability, and 3 points for exposure or outcome. According to the NOS, We classified the qualities of these studies into three groups: high (7–9 points), intermediate (4–6 points) and low (1–3 points) qualities [27].

### 4 Data extraction

Two independent researchers (R.-x.T. and T.-s.Z.) reviewed all of the articles that met the inclusion criteria. We selected OS as the primary endpoints, RFS and EFS as the secondary endpoints [6], [28]. We extracted characteristics of the studies, the corresponding HRs with 95% CIs from COX multivariable models. Unpublished data in some studies were obtained by contacting the authors. If the HRs with 95% CIs were not presented in some articles, we would extract the data from corresponding Kaplan-Meier curves using the methods described by Parmar and Tierney [29], [30].

### 5 Data synthesis and statistical analysis

We pooled the HRs and 95% CIs for OS, RFS and EFS respectively in total population and subgroups, using random-effect and fixed-effect models simultaneously [31], but only the estimates from random effects model was selected as the basis of our conclusion, because this approach could provide a more conservative assessment of the average effect size [32]. In the forest plots, pooled HR values>1 represented a positive association between mutant DNMT3A and prognosis of patients; while pooled HR values<1 represented a negative association.

### 6 Assessment of heterogeneity

We assessed statistical heterogeneity as to the pooled HR for OS in total population by visual inspection of the forests plots and by a formal statistical test using Chi-square test with a significance level at P<0. 1. I^2^ statistics was also used to quantitatively assess the possible heterogeneity (I^2^>30% represents moderate heterogeneity, I^2^>75% represents considerable heterogeneity) [33].

### 7 Subgroup analysis

We explored the possible causes of heterogeneity by subgroups analysis. We examined the following as important subgroups: (1) cytogenetic intermediate-risk subgroup; (2) cytogenetically normal subgroup; (3) patients younger than 60 years of age; (4) patients older than 60 years of age. (5) favorable risk genotype subgroup, namely low risk molecular group, which referred to the patients with mutant NPM1 and wild-type FLT3-ITD (NPM1+/FLT3-ITD−); (6) unfavorable risk genotype subgroup, also known as high risk molecular group, which included the patients (NPM1−/FLT3-ITD+, NPM1−FLT3-ITD− or NPM1+/FLT3-ITD+); (7) patients with DNMT3A mutations at R882; and (8) patients with DNMT3A mutations at Non-R882. Of note, NPM1/FLT3-ITD risk genotype groups were all belong to CN-AML.

### 8 Assessment of reporting biases and sensitivity analysis

Sensitivity analysis was performed to detect the robustness of pooled HR for OS of all patients. Firstly, to test for publication bias, We selected OS of total population to form a funnel plot [34], [35] and contour enhanced funnel plot [36], and undertook the Egger test as well. Different types of funnel plots were presented in a similar form of scatter plots of study effect sizes (x axis) against estimated standard errors (y axis). Contour enhanced funnel plots display areas of statistical significance on a funnel plot. Contour lines represent different levels of statistical significance (e. g. 5%, 10%). If studies seemed to be missing in areas of non-significance (P>10%), the asymmetry of the funnel plot might be due to publication bias, although other explanations should still be considered. Meanwhile, we attempted to find other reporting biases such as outcome reporting bias, language bias, citation bias and location bias and so on.

In addition, we tried to figure out “missing” studies and assessed the robustness of pooled HR for OS of the overall population by combining contour enhanced funnel plots with the trim and fill adjustment method(random and fixed effects linear estimator) [37], [38], [39], All statistical analyses were performed by the software STATA (version 12. 0).

## Results

### 1 Search results

**Figure 1:**
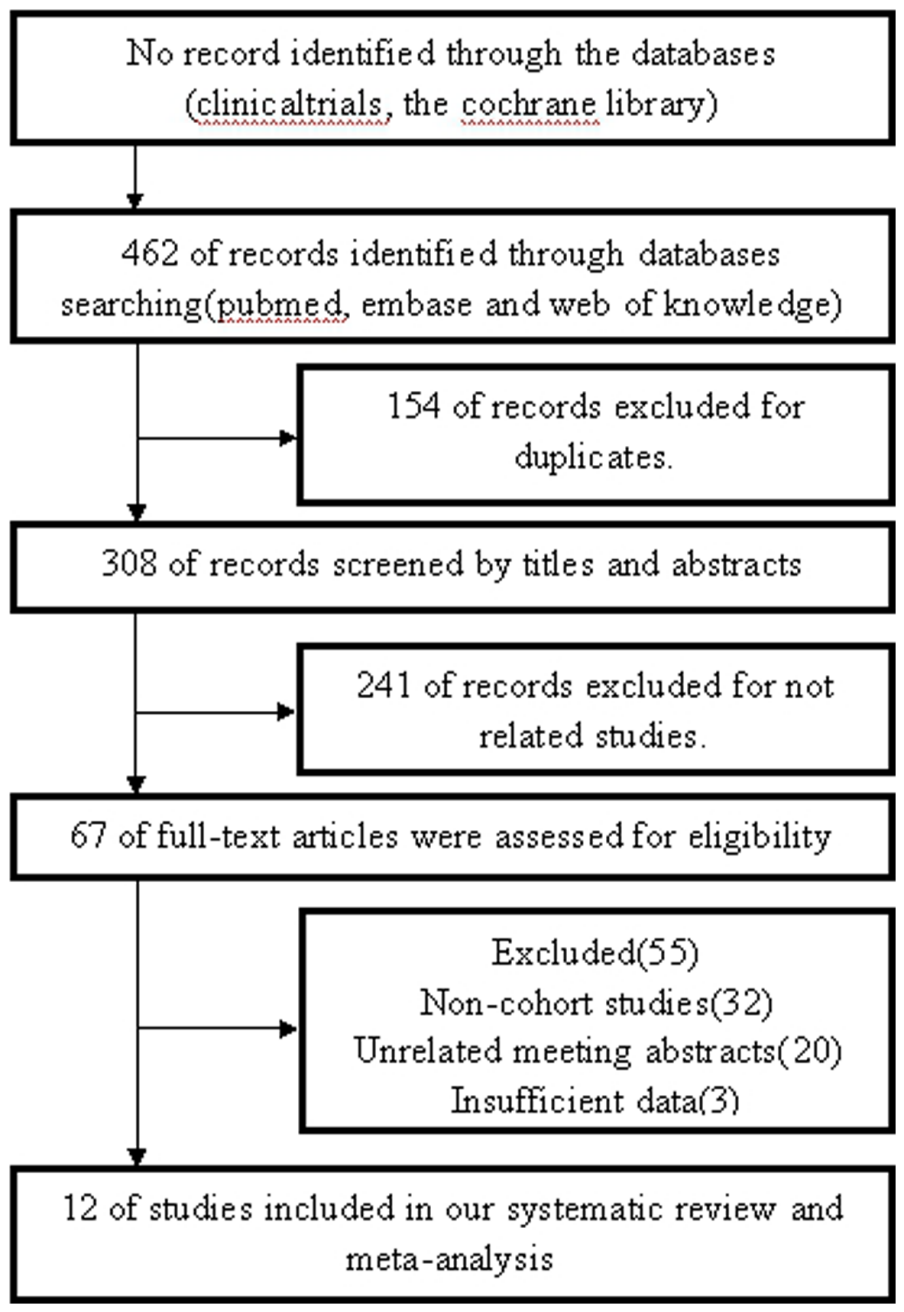
PRISMA flow diagram for study review and inclusion.

As shown in **Figure 1**, 462 records were obtained by searching the databases. After exclusion of 154 duplicates, 308 records were further screened by reading their titles and abstracts, and then 241 records were excluded, in which DNMT3A mutations were not regarded as the primary research problems. The full texts of the remaining 67 studies were read carefully, and 55 of them including 32 non-cohort studies, 20 unrelated meeting abstracts and 3 studies having no sufficient data were further excluded. Eventually, 12 studies met the predefined selection criteria, which included 2 meeting abstract [40] and 10 original articles [13], [20], [21], [22], [23], [24], [25], [41], [42], [43].

### 2 Risk of bias in the included studies

We assessed risk of bias (see methodological quality domains), according to nine items of NOS. The qualities of 9 (75%) studies were considered high, and the remaining 3 (25%) studies were thought moderate. More details were shown in **Table 1** and **Table 2**.

**Table 1:** Clinical and laboratory characteristics of patients with AML with DNMT3A mutations from the 12 included studies.

First author	Year	Source	NOS	n/N	Median follow-up, mo (range)	Median age, y (range)	FAB classification	Cytogenetic risk group	HR estimation
Timothy J. Ley	2010	USA	9	62/281	34.1 (0.2–129.3)	53.1 (39.4–66.8)	M0, 2; M1, 15; M2, 11; M3, 1; M4, 20; M5, 12; M6, 0; M7, 1; RAEB, 0; Unknown, 0	Favorable, 0; Intermediate, 56; Adverse, 4; Unknown, 2	HR
Yang Shen	2011	China	7	75/1141	—	38–69	—	—	HR
Felicitas Thol	2011	Germany	9	87/489	61.2 (0.624–140.4)	52 (30–60)	M0, 3; M1, 15; M2, 8; M3, 0; M4, 37; M5, 22; M6, 1; M7, 0; RAEB, —; Unknown, 1	Favorable, 1; Intermediate, 82; Adverse, 4; Unknown, 0	HR
Hsin-An Hou	2011	Taiwan	8	70/500	55 (1.0–160)	61 (16–87)	M0, 2; M1, 14; M2, 13; M3, 0; M4, 28; M5, 12; M6, 0; M7, —; RAEB, —; Unknown, 1	Favorable, 0; Intermediate, 62; Adverse, 4; Unknown, 4	HR
Jana Markova	2011	Czech Republic	5	67/226	11.6 (0–202)	54.9 (18.2–81.7)	M0, 0; M1, 18; M2, 22; M3, —; M4, 17; M5, 5; M6, —; M7, —; RAEB, —; Unknown, 5	Favorable, 0; Intermediate, 67; Adverse, 0; Unknown, 0	Survival curve
A Renneville	2012	France	7	36/123	46.8	47 (23–58)	M0, 0; M1, 6; M2, 10; M3, 0; M4, 11; M5, 9; M6, 0; M7, 0; RAEB, 0; Unknown, 0	Favorable, 0; Intermediate, 36; Adverse, 0; Unknown, 0	HR
Guido Marcucci	2012	USA	9	142/415	90 (27.6–488)	61 (22–82)	M0, 1; M1, 29; M2, 18; M3, 0; M4, 33; M5, 26; M6, 0; M7, —; RAEB, —; Unknown, 35	Favorable, 0; Intermediate, 142; Adverse, 0; Unknown, 0	HR
Jay P. Patel	2012	USA	9	88/384	—	48 (17–60)	—	—	HR
Ana Flávia Tibúrcio Ribeiro	2012	USA	9	96/415	115.7 (7.2–224.1)	50.5 (18–60)	M0, 1; M1, 15; M2, 23; M3, —; M4, 15; M5, 36; M6, 1; M7, —; RAEB, 3; Unknown, 2	Favorable, 0; Intermediate, 85; Adverse, 6; Unknown, 5	HR
Xu, Y	2012	China	5	31/442	—	40 (16–60)	—	—	HR
Ostronoff, F	2013	USA	6	37/191	101.6	68 (57–81)	M0, 2; M1, 9; M2, 6; M3, 0; M4, 10; M5, 7; M6, 0; M7, 0; RAEB, 0; Unknown, 3	Favorable, 0; Intermediate, 24; Adverse, 1; Unknown, 12	HR
Verena I. Gaidzik	2013	Germany	9	370/1770	59.28	50.5 (18–60)	—	Favorable, 4; Intermediate, 309; Adverse, 33; Unknown, 24	HR

NOS, Newcastle-ottawa quality assessment scale; n, number of patients with DNMT3A mutations; N, number of patients in total; —, indicates there is no related data presented.

**Table 2:** Clinical and laboratory characteristics of patients with AML without DNMT3A mutations from the 12 included studies.

First author	Year	Source	NOS	(N-n)/N	Median follow-up, mo (range)	Median age, y (range)	FAB classification	Cytogenetic risk group	HR estimation
Timothy J. Ley	2010	USA	9	219/281	34.1 (0.2–129.3)	47.8 (31.2–64.4)	M0, 19; M1, 44; M2, 54; M3, 47; M4, 41; M5, 9; M6, 3; M7, 2; RAEB, 0; Unknown, 0	Favorable, 79; Intermediate, 110; Adverse, 26; Unknown, 4	HR
Yang Shen	2011	China	7	1066/1141	—	18.6–59.4	—	—	HR
Felicitas Thol	2011	Germany	9	402/489	61.2 (0.624–140.4)	45 (17–60)	M0, 7; M1, 74; M2, 113; M3, 0; M4, 130; M5, 44; M6, 11; M7, 3; RAEB, —; Unknown, 20	Favorable, 78; Intermediate, 262; Adverse, 57; Unknown, 5	HR
Hsin-An Hou	2011	Taiwan	8	430/500	55 (1.0–160)	49 (15–90)	M0, 8; M1, 98; M2, 158; M3, 38; M4, 96; M5, 12; M6, 12; M7, —; RAEB, —; Unknown, 8	Favorable, 99; Intermediate, 256; Adverse, 61; Unknown, 14	HR
Jana Markova	2011	Czech Republic	5	159/226	11.6 (0–202)	54.9 (18.2–81.7)	M0, 7; M1, 29; M2, 46; M3, —; M4, 33; M5, —; M6, 12; M7, —; RAEB, —; Unknown, 32	Favorable, 0; Intermediate, 159; Adverse, 0; Unknown, 0	Survival curve
A Renneville	2012	France	7	87/123	46.8	48 (16–59)	M0, 5; M1, 24; M2, 27; M3, —; M4, 11; M5, 7; M6, 7; M7, —; RAEB, —; Unknown, 6	Favorable, 0; Intermediate, 87; Adverse, 0; Unknown, 0	HR
Guido Marcucci	2012	USA	9	273/415	90 (27.6–488)	62 (18–83)	M0, 7; M1, 54; M2, 71; M3, 0; M4, 41; M5, 21; M6, 3; M7, —; RAEB, —; Unknown, 76	Favorable, 0; Intermediate, 273; Adverse, 0; Unknown, 0	HR
Jay P. Patel	2012	USA	9	296/384	—	48 (17–60)	—	—	HR
Ana Flávia Tibúrcio Ribeiro	2012	USA	9	319/415	115.7 (7.2–224.1)	41 (15–60)	M0, 15; M1, 72; M2, 81; M3, —; M4, 64; M5, 64; M6, 5; M7, —; RAEB, 13; Unknown, 5	Favorable, 57; Intermediate, 191; Adverse, 64; Unknown, 7	HR
Xu, Y	2012	China	5	411/442	—	40 (16–60)	—	—	HR
Ostronoff, F	2013	USA	6	154/191	101.6	68 (56–89)	M0, 3; M1, 35; M2, 55; M3, 1; M4, 38; M5, 13; M6, 2; M7, 5; RAEB, 0; Unknown, 3	Favorable, 12; Intermediate, 54; Adverse, 32; Unknown, 56	HR
Verena I. Gaidzik	2013	Germany	9	1400/1770	59.28	47.6 (18–60)	—	Favorable, 252; Intermediate, 751; Adverse, 287; Unknown, 110	HR

NOS, Newcastle-ottawa quality assessment scale; n, number of patients with DNMT3A mutations; N, number of patients in total; —, indicates there is no related data presented.

### 3 Characteristics of the included studies

The main characteristics of the patients with or without DNMT3A mutations in 12 included studies were presented in **Table 1** and **Table 2** respectively, additional characteristics of the patients were shown in **Tables S1** and **S2**. Of these studies, 5 originated from America, 4 from Europe and 3 from Asia.

### 4 Data extracting

We obtained directly most of data from the included studies except for one set of data [25], which were extracted indirectly from the corresponding Kaplan-Meier curves related to mutant DNMT3A. Additionally, we managed to obtain some unpublished data by contacting the authors. Hou et al. offered the HRs with 95% CIs for OS and RFS in unfavorable and favorable risk genotype subgroups with AML. Fabiana et al. offered the HRs with 95% CIs for OS, EFS and RFS in subgroups as mentioned above.

### 5 Association of DNMT3A mutations with pretreatment clinical and molecular characteristics

More patients with DNMT3A mutations were found with mutations in NPM1, FLT3-ITD and IDH1/2 [13], [20], [21], [22], [23], [24], [42], whereas CEBPA mutations hardly coexisted in the patients with DNMT3A mutations [20], [21], [22], [42], [43]. DNMT3A mutations were mainly enriched in the patients with CN-AML or with intermediate-risk AML. Patients with favorable-risk cytogenetics had scarcely DNMT3A mutations. Comparing with wild-type DNMT3A patients, DNMT3A mutated patients were older, with higher median WBC counts and percentage of bone marrow blasts at diagnosis. Whereas, platelet counts of patients were not considered to be associated with DNMT3A mutations in most of the included studies [21], [22], [23], [24].

### 6 Association of DNMT3A mutations with clinical outcomes in total population

**Figure 2:**
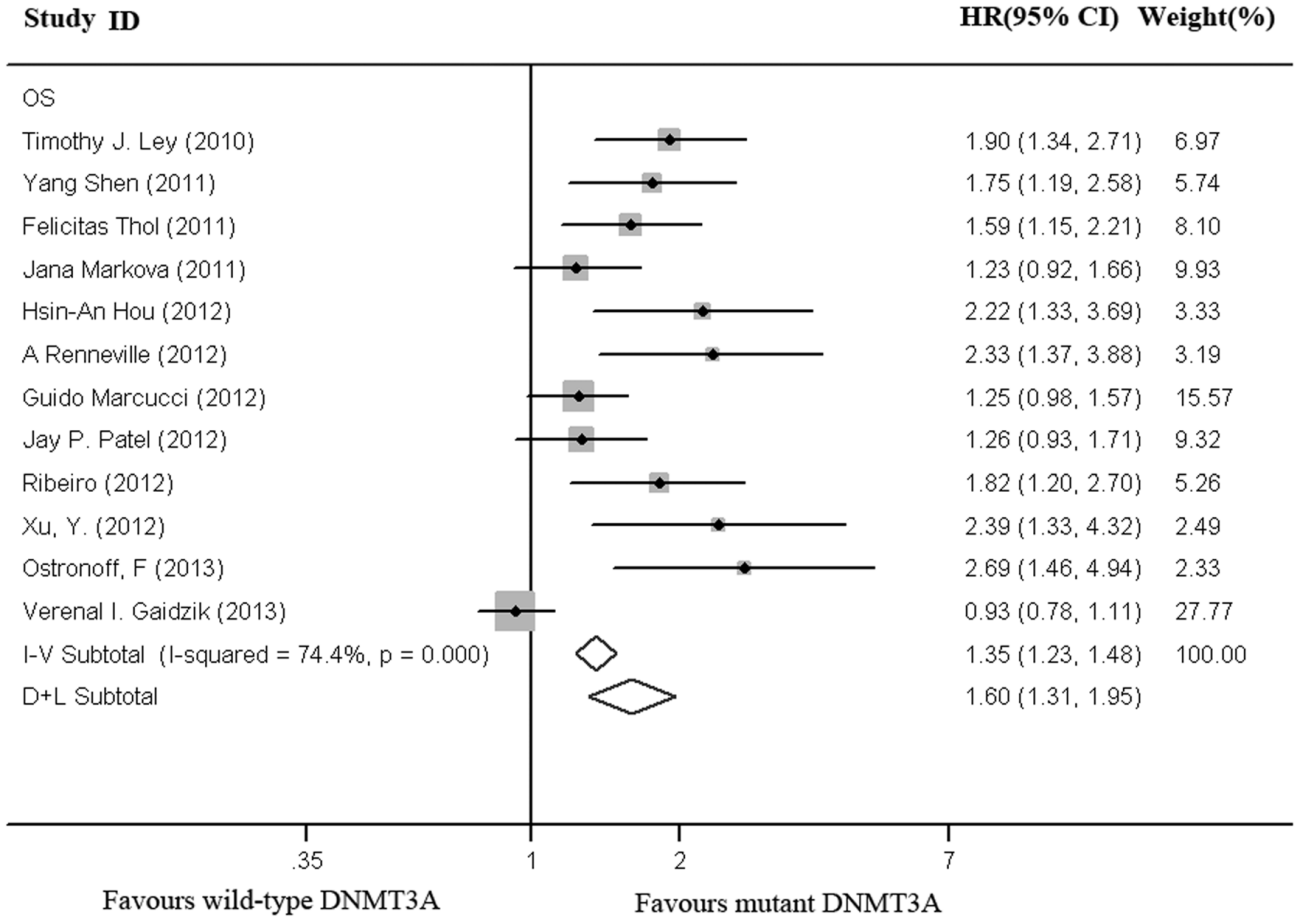
Forest plot of the HR for overall survival of all AML patients. DNMT3A mutations versus wild-type DNMT3A. I–V Overall: the pooled HR with 95% CI using a fixed effects model; D+L Overall: the pooled HR with 95% CI using a random effects model.

As shown in **Figure 2**, data were extracted from 12 studies, with a total of 6377 patients, including 1161 patients with DNMT3A mutation. In the total population, the patients with DNMT3A mutations had a shorter OS as compared to those with wild-type DNMT3A (HR = 1.60, 95% CI, 1.31–1.95). There was considerable heterogeneity as shown by I^2^ testing (I^2^ = 74.4%, P<0. 001).

**Figure 3:**
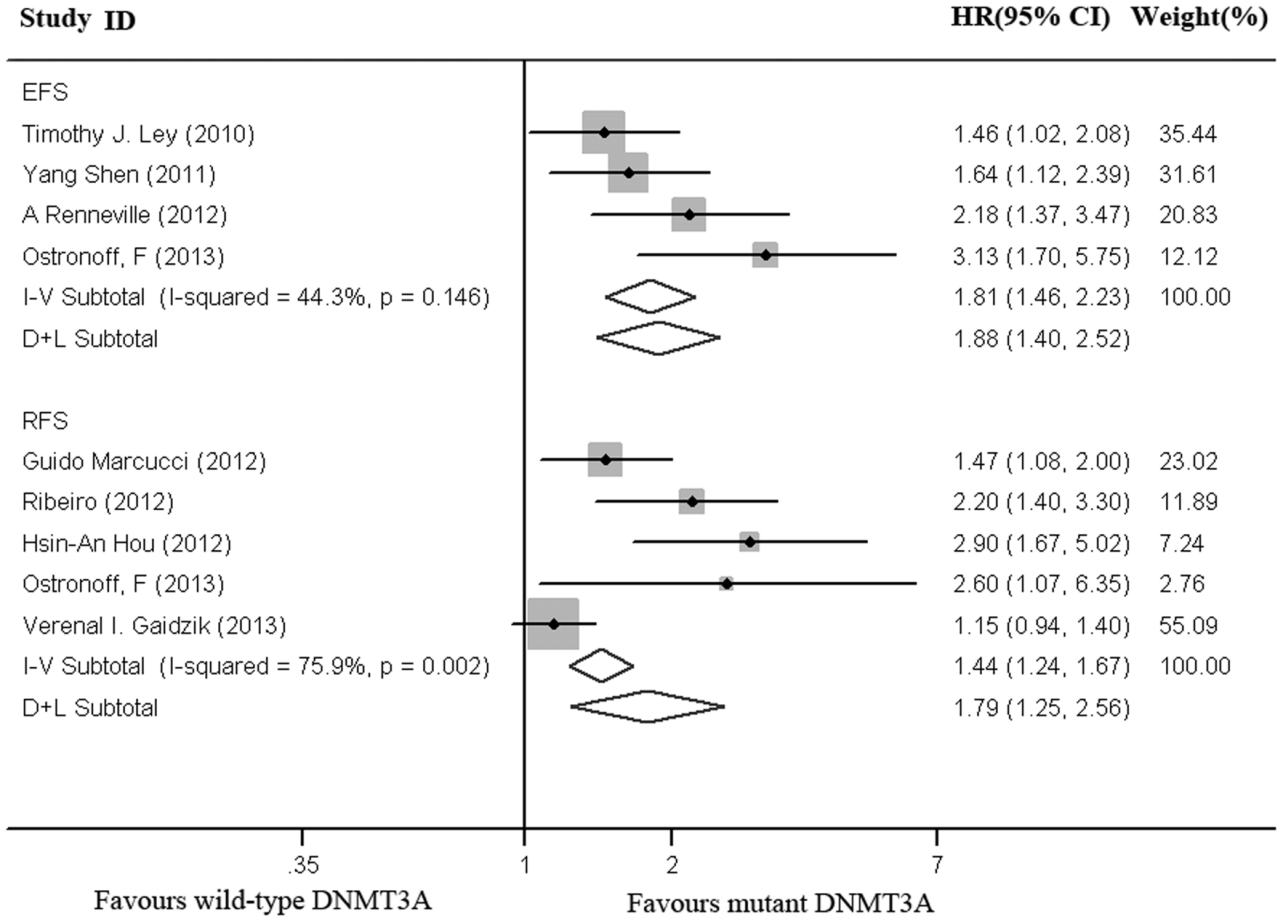
Forest plot of the HRs for relapse-free or event-free survival of AML patients. DNMT3A mutations versus wild-type DNMT3A. I–V Subtotal represented the pooled HRs with 95% CIs using a fixed effects model; D+L Subtotal represented the pooled HR with 95% CI using a random effects model.

As shown in **Figure 3**, data were extractable from 5 out of 12 studies, with a total of 3291 patients, including 715 DNMT3A mutated patients. In this population, patients with DNMT3A mutations had a shorter RFS as compared to those with wild-type DNMT3A (HR = 1.79, 95% CI, 1.25–2.56). There was considerable heterogeneity by I^2^ testing (I^2^ = 75.9%, P = 0.002).

As shown in **Figure 3**, data were extractable from 4 out of 12 studies, with a total of 1736 patients, including 210 DNMT3A mutated patients. In this population, patients with DNMT3A mutations had a shorter EFS as compared to them with wild-type DNMT3A (HR = 1.88, 95% CI, 1.40–2.52). There was moderate heterogeneity by I^2^ testing (I^2^ = 44.3%, P = 0.146).

### 7 Association of DNMT3A mutations with clinical outcomes in different subgroups

We pooled the HRs in fixed- and random-effect models simultaneously, which were shown in **Table 3** and **Table 4** respectively, corresponding forest plots were presented in **Figures S1** to **S8**. In the favorable risk genotype subgroup, mutant DNMT3A had no prognostic impact on OS or RFS other than EFS. And in the non-R882-DNMT3A mutations subgroup, mutant DNMT3A had no prognostic value for OS. However, DNMT3A mutations were independently prognostic factors for OS, RFS and EFS of patients in the remaining 6 subgroups. There was considerable statistical heterogeneity detected by I^2^ test in most of these subgroups.

**Table 3:** Outcome of subgroups in a random effects model.

Subgroups	OS					RFS					EFS				
	N. of S.	D+/−	I^2^ (%)	HR (95% CI)	P	N. of S.	D+/−	I^2^ (%)	HR (95% CI)	P	N. of S.	D+/−	I^2^ (%)	HR (95% CI)	P
CN-AML	7	760/2321	74.4	1.60 (1.20–2.13)	0.001	5	562/1582	67.3	1.68 (1.20–2.36)	0.016	2	99/373	62.1	2.43 (1.18–5.00)	0.104
Intermediate-risk cytogenetics	3	225/697	67.2	1.51 (1.08–2.11)	0.047	2	183/721	0.0	1.52 (1.19–1.93)	0.937	0	—	—	—	—
Younger than 60 years of age	10	1054/4836	80.7	1.84 (1.36–2.50)	<0.001	4	597/1971	84.3	2.10 (1.20–3.69)	<0.001	3	173/1372	0.0	2.32 (1.78–3.02)	0.949
Unfavorable risk genotype	4	230/1073	0.0	2.30 (1.78–2.97)	0.862	3	194/986	0.0	2.38 (1.69–3.36)	0.726	2	73/241	0.0	1.74 (1.17–2.60)	0.78
Favorable risk genotype	6	388/1611	0.0	1.40 (0.98–1.99)	0.798	4	290/1305	41.1	1.18 (0.62–2.24)	0.165	2	73/241	0.0	1.99 (1.09–3.61)	0.432
Older than 60 years of age	2	204/492	0.0	1.71 (1.27–2.31)	0.759	0	—	—	—	—	0	—	—	—	—
R882-DNMT3A-mt/DNMT3A-wt	4	539/2144	74.5	1.41 (1.06–1.88)	0.008	3	432/1541	0.0	1.36 (1.16–1.61)	0.757	0	—	—	—	—
Non-R882-DNMT3A-mt/DNMT3A-wt	3	332/1825	89	1.25 (0.69–2.25)	<0.001	0	—	—	—	—	0	—	—	—	—

N.of S., number of studies;

D+/−, indicates ratio of number of patients with mutant DNMT3A to patients with wild-type DNMT3A;

—, reflects there is no corresponding data presented; Abbreviations: mt, mutation; wt, wild type.

**Table 4:** Outcome of subgroups in a fixed effects model.

Subgroups	OS					RFS					EFS				
	N. of S.	D+/−	I^2^ (%)	HR (95% CI)	P	N. of S.	D+/−	I^2^ (%)	HR (95% CI)	P	N. of S.	D+/−	I^2^ (%)	HR (95% CI)	P
CN-AML	7	760/2321	74.4	1.33 (1.17–1.52)	0.001	5	562/1582	67.3	1.44 (1.22–1.71)	0.016	2	99/373	62.1	2.19 (1.46–3.27)	0.104
Intermediate-risk cytogenetics	3	225/697	67.2	1.45 (1.20–1.75)	0.047	2	183/721	0.0	1.52 (1.19–1.93)	0.937	0	—	—	—	—
Younger than 60 years of age	10	1054/4836	80.7	1.40 (1.25–1.58)	<0.001	4	597/1971	84.3	1.44 (1.22–1.71)	<0.001	3	173/1372	0.0	2.32 (1.78–3.02)	0.949
Unfavorable risk genotype	4	230/1073	0.0	2.30 (1.78–2.97)	0.862	3	194/986	0.0	2.38 (1.69–3.36)	0.726	2	73/241	0.0	1.74 (1.17–2.60)	0.78
Favorable risk genotype	6	388/1611	0.0	1.40 (0.98–1.99)	0.798	4	290/1305	41.1	1.19 (0.75–1.88)	0.165	2	73/241	0.0	1.99 (1.09–3.61)	0.432
Older than 60 years of age	2	204/492	0.0	1.71 (1.27–2.31)	0.759	0	—	—	—	—	0	—	—	—	—
R882-DNMT3A-mt/DNMT3A-wt	4	539/2144	74.5	1.30 (1.14–1.50)	0.008	3	432/1541	0.0	1.36 (1.16–1.61)	0.757	0	—	—	—	—
Non-R882-DNMT3A-mt/DNMT3A-wt	3	332/1825	89	1.08 (0.90–1.31)	<0.001	0	—	—	—	—	0	—	—	—	—

N.of S., number of studies;

D+/−, indicates ratio of number of patients with mutant DNMT3A to patients with wild-type DNMT3A;

—, reflects there is no corresponding data presented; Abbreviations: mt, mutation; wt, wild type.

### 8 Publication bias and sensitive analysis

We utilized the data about OS of total population to search publication bias and do sensitive analysis.

**Figure 4:**
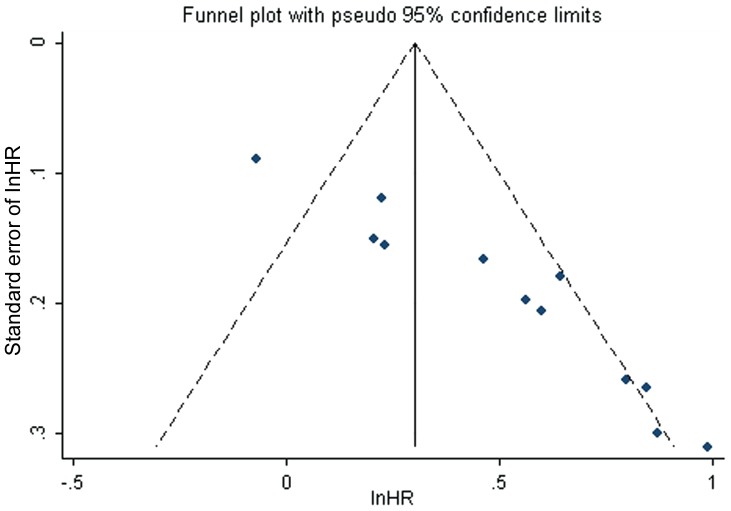
Funnel plots illustrated significant asymmetry on HR for overall survival of all patients. Studies were distributed asymmetrically and suggested biases exist.

As shown in **Figure 4**, there was a strong suggestion of asymmetry in the funnel plot (Egger's test P<0. 001).

**Figure 5:**
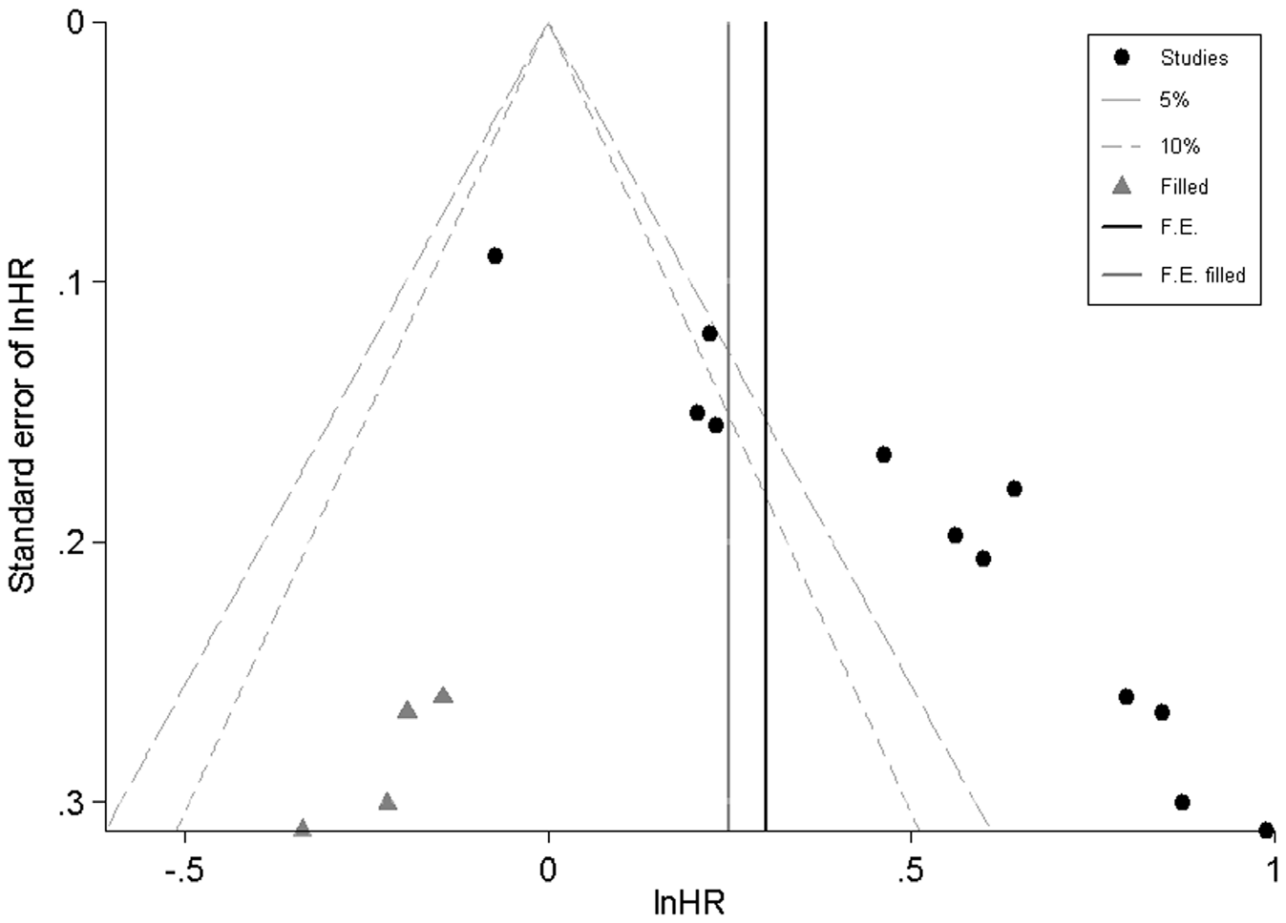
Confunnel with filled studies from metatrim: mutant DNMT3A versus wild-type DNMT3A in a random effects model. The pooled HR on overall survival from 12 published studies is robust and the heterogeneity mainly results from unpublished studies.

**Figure 5** demonstrated the distribution of 12 real studies and 4 filled studies. Of the 12 real studies, 8 studies lay above the right 5% significant contour line, and 4 studies located below the 5% significant contour line. Only one study distributed on the left-hand side of the plot, which also implied asymmetry of the plot. Secondly, the trim and fill method imputed a total of 4 “missing” studies in a random effects model, which were presented in the form of gray triangles located in the region of statistical non-significance (P>10%), and the vertical black line showed the pooled log HR as to OS in 12 studies, while the vertical gray line showed the pooled log HR including the filled studies. After adding the “missing” studies to the contour-enhanced funnel plot, the pooled fixed-effects HR was 1.282 (95% CI, 1.174–1.400), and the pooled random-effects HR was 1.394 (95% CI, 1.161–1.674), which implied DNMT3A mutations had a slightly negative impact on OS in total population.

## Discussion

We have performed a systematic review and meta-analysis in an effort to figure out the association between DNMT3A mutations and poor prognosis of patients with de novo AML.

So far, the 12 published studies have addressed the prognostic value of mutant DNMT3A in AML patients. According to the rules of NOS, the majority of included studies belonged to high quality, only 3 of the studies had moderate quality. A total of 6377 patients of the 12 included studies, where there were 1161 patients with mutant DNMT3A.

The primary end point was OS. DNMT3A mutations had slightly prognostic impact on OS in total population, which was consistent with the pooled outcomes in the meta-analysis by Shivarov et al [44]. And the conclusion was suitable for the majority of subgroups except for the favorable risk genotype and non-R882 DNMT3A mutation subgroups. The second endpoints were RFS and EFS. First of all, mutant DNMT3A had poor prognostic impact on RFS in nearly all of the mentioned groups other than favorable risk genotype subgroup. As for EFS, DNMT3A mutations were associated with poor prognosis of patients in all of the mentioned groups above. However, most of the included studies showed neither type of DNMT3A mutation had an impact on the probability of achieving CR in multivariate analyses [21], [22], [23], [24], [41], [42].

There were significant correlation of DNMT3A mutations with patient characteristics, including age of patients and WBC in peripheral blood at diagnosis, the mutations of NPM1, FLT3 or other genes (IDH1, IDH2, TET2, WT1 and CEBPA), regardless of the types of mutation. In addition, only one study mentioned the association between DNMT3A mutations and expression of CD molecules on leukemic initiating cells, such as CD14, CD13 and CD34 [24].

We observed considerable or moderate between-study heterogeneity in total population and subgroups, unlike that in the meta-analysis by Shivarov et al [44].

The heterogeneity mainly came from one study lately published [26]. It was worth noting that we did not conduct a meta-regression analysis because of fewer studies included. So we explored subjectively the clinical heterogeneity as follows: firstly, with respect to FAB classification, patients' years of age, cytogenetic and molecular abnormalities, the constituent ratio of patients were various in each study, for instance, 7 studies only included patients younger than 60 years of age [20], [21], [23], [40], [41], [42], 2 studies only included cytogenetic normal patients [22], [42], 1 study included patients belonging to cytogenetic intermediate risk group [22]; secondly, most of the included studies did not presented the ECOG performance status of patients except for two of them [23], [43], so we could not further assess the status of patients when they were included, which might be one of the sources of heterogeneity; thirdly, the treatment programs such as induced chemotherapy and hematopoietic stem cell transplantation were diverse in individual studies, even intensities of the same induced chemotherapy drug were various in different studies, so the selection of different treatment programs might lead to the heterogeneity of clinical outcomes; eventually, there were much between-study heterogeneity in other aspects such as the time of follow up, percentages of bone marrow blasts, races of patients, types of DNMT3A mutations, the methods of gene sequencing, and the number of adjusted covariates among the 12 studies and so on.

We found the asymmetry in a usual funnel plot. Subsequently, we adjusted pooled HR for OS in total population by combining the trim and fill method with contour enhanced funnel plot, and found that the asymmetry was probably caused by unpublished “missing” studies, so publication bias was a major threat to the validity of our estimate outcomes, although other explanations should still be considered. Meanwhile, we verified the robustness of the pooled random-effect and fixed-effect outcomes of 16 studies consistent with the pooled outcomes of 12 original studies, which was extremely important for our eventual conclusion.

We did not find any methodological issues in the preparation of the review which could put it at risk for bias.

There are the following aspects of main limitation in our review. Firstly, the “missing” studies have been relatively absent to date. Secondly, selective reporting were seen in some studies, for instance, the pooled HRs for RFS and EFS were shown in relatively fewer studies as compared to them for OS, and some subgroups such as older patients were not analyzed in most studies, which resulted in losses of valuable information. Thirdly, two studies were published as meeting abstracts, so we could not assess its potential risk of bias in detail. Fourthly, the included studies were all published in English, so language bias existed. Fifthly, we could not access the association between mutant DNMT3A and prognosis of secondary AML because of the lack of efficient data. Finally, if these trials could offer individual patient data, our analysis would have been much more perfect.

In conclusion, using some advanced statistical methods and high quality evidences, we confirmed objectively the robustness of association between mutant DNMT3A and prognosis of adults with de novo AML, although there are considerable heterogeneity in many conditions. Combining other important genetic biomarkers, DNMT3A mutations would contribute to a more precise clinical risk stratification and decision of treatment. More cohort studies concerning DNMT3A mutations are needed in an effort to further verify or modify the pooled estimates to a certain extent, especially for the patients with CN-AML or with cytogenetic intermediate risk abnormalities. It is meaningful to clarify the mechanism that mutant DNMT3A function as a epigenetic regulator in adult AML. And more studies will be needed to determine whether dose-intensified induction chemotherapy improves the survival outcomes in patients with DNMT3A mutations.

## Supporting Information

Figure S1 **Forest plot of the HRs with 95% CIs for OS, RFS and EFS of CN-AML patients (mutant DNMT3A versus wild-type DNMT3A).**
(TIF)

Figure S2 **Forest plot of the HRs with 95% CIs for OS and RFS of AML patients with intermediate-risk cytogenetics (mutant DNMT3A versus wild-type DNMT3A).**
(TIF)

Figure S3 **Forest plot of the HRs with 95% CIs for OS, RFS and EFS of patients younger than 60 years of age (mutant DNMT3A versus wild-type DNMT3A).**
(TIF)

Figure S4 **Forest plot of the HRs with 95% CIs for OS, RFS and EFS of patients with unfavorable risk genotype (mutant DNMT3A versus wild-type DNMT3A).**
(TIF)

Figure S5 **Forest plot of the HRs with 95% CIs for OS, RFS and EFS of patients with favorable risk genotype (mutant DNMT3A versus wild-type DNMT3A).**
(TIF)

Figure S6 **Forest plot of the HR with 95% CI for OS of patients older than 60 years of age (mutant DNMT3A versus wild-type DNMT3A).**
(TIF)

Figure S7 **Forest plot of the HRs with 95% CIs for OS and RFS of patients with R882-mutant DNMT3A as compared to patients with wild-type DNMT3A.**
(TIF)

Figure S8 **Forest plot of the HR with 95% CI for OS of patients with Non-R882-mutant DNMT3A as compared to patients with wild-type DNMT3A.**
(TIF)

Table S1 **Clinical and laboratory characteristics of patients with AML with DNMT3A mutations from the 12 included studies.**
(DOCX)

Table S2 **Clinical and laboratory characteristics of patients with AML without DNMT3A mutations from the 12 included studies.**
(DOCX)

Checklist S1 **The PRISMA 2009 checklist.**
(DOC)
