# Supplementary figures and images for: Association between DNMT3A Mutations and Prognosis of Adults with De Novo Acute Myeloid Leukemia: A Systematic Review and Meta-Analysis
Source: PLoS One. 2014 Jun 17;9(6):e93353. doi: 10.1371/journal.pone.0093353 (PMC4061003; doi:10.1371/journal.pone.0093353)

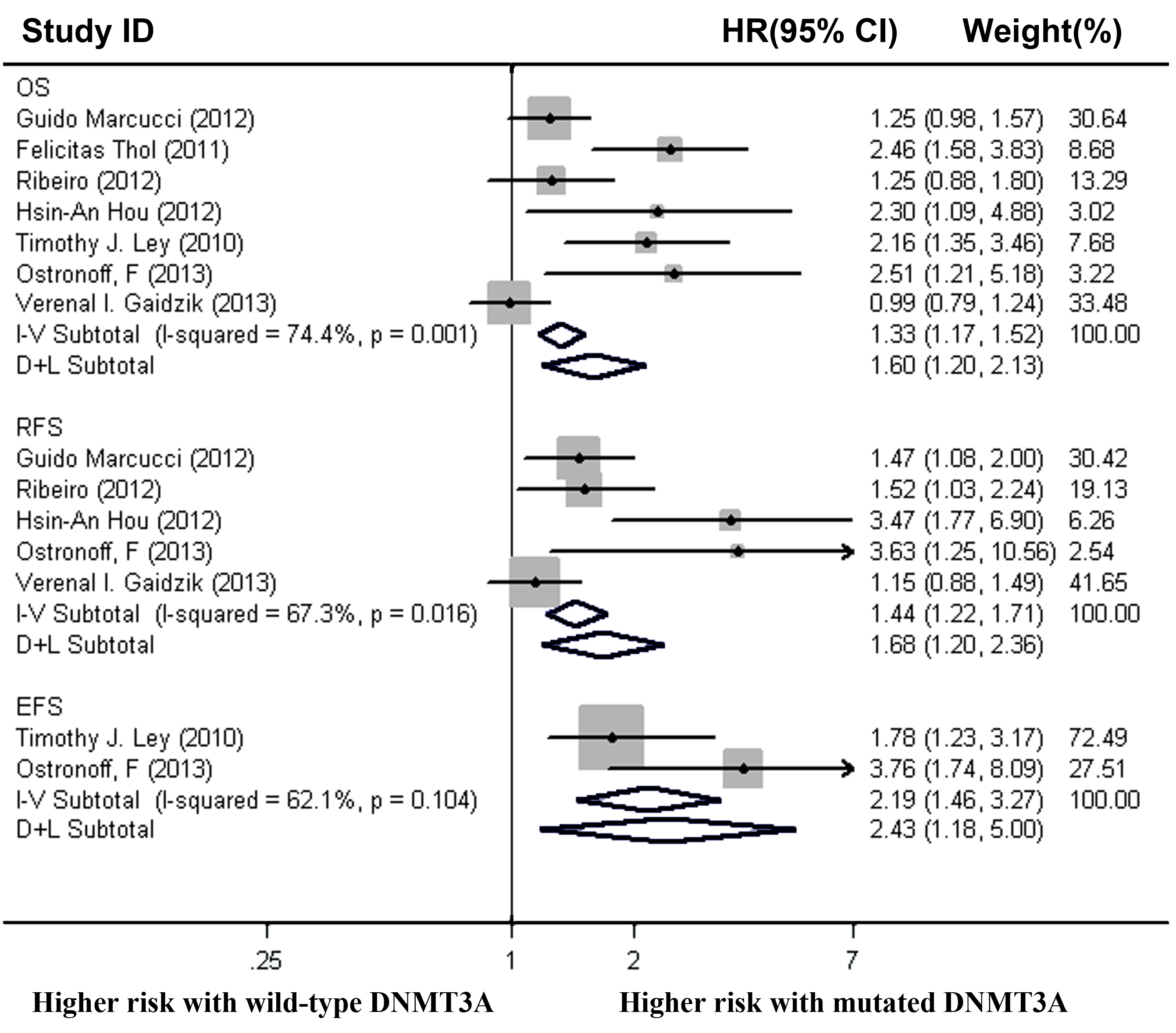

Supplement: Figure S1 — Forest plot of the HRs with 95% CIs for OS, RFS and EFS of CN-AML patients (mutant DNMT3A versus wild-type DNMT3A). (TIF) [file pone.0093353.s001.tif]

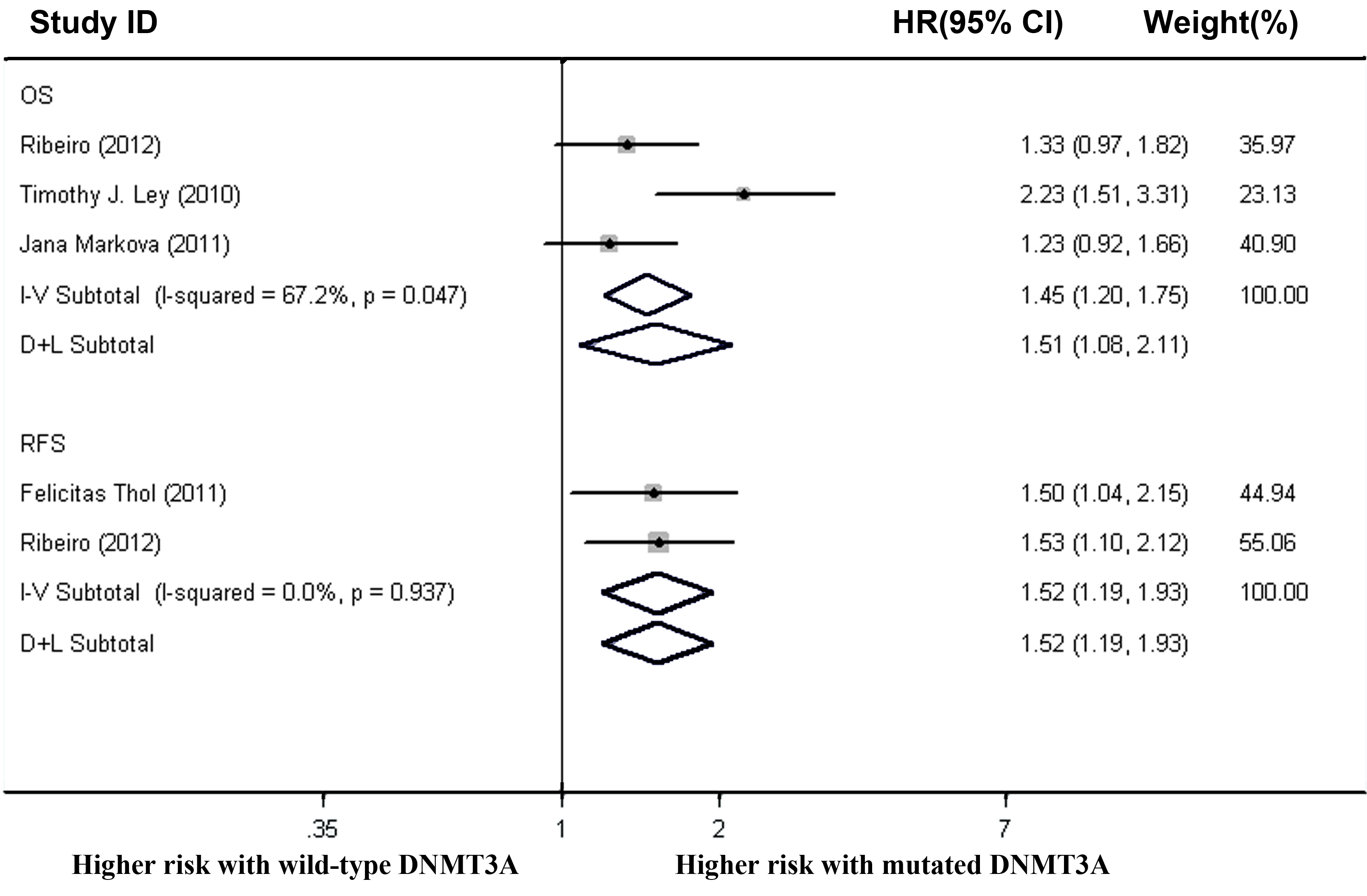

Supplement: Figure S2 — Forest plot of the HRs with 95% CIs for OS and RFS of AML patients with intermediate-risk cytogenetics (mutant DNMT3A versus wild-type DNMT3A). (TIF) [file pone.0093353.s002.tif]

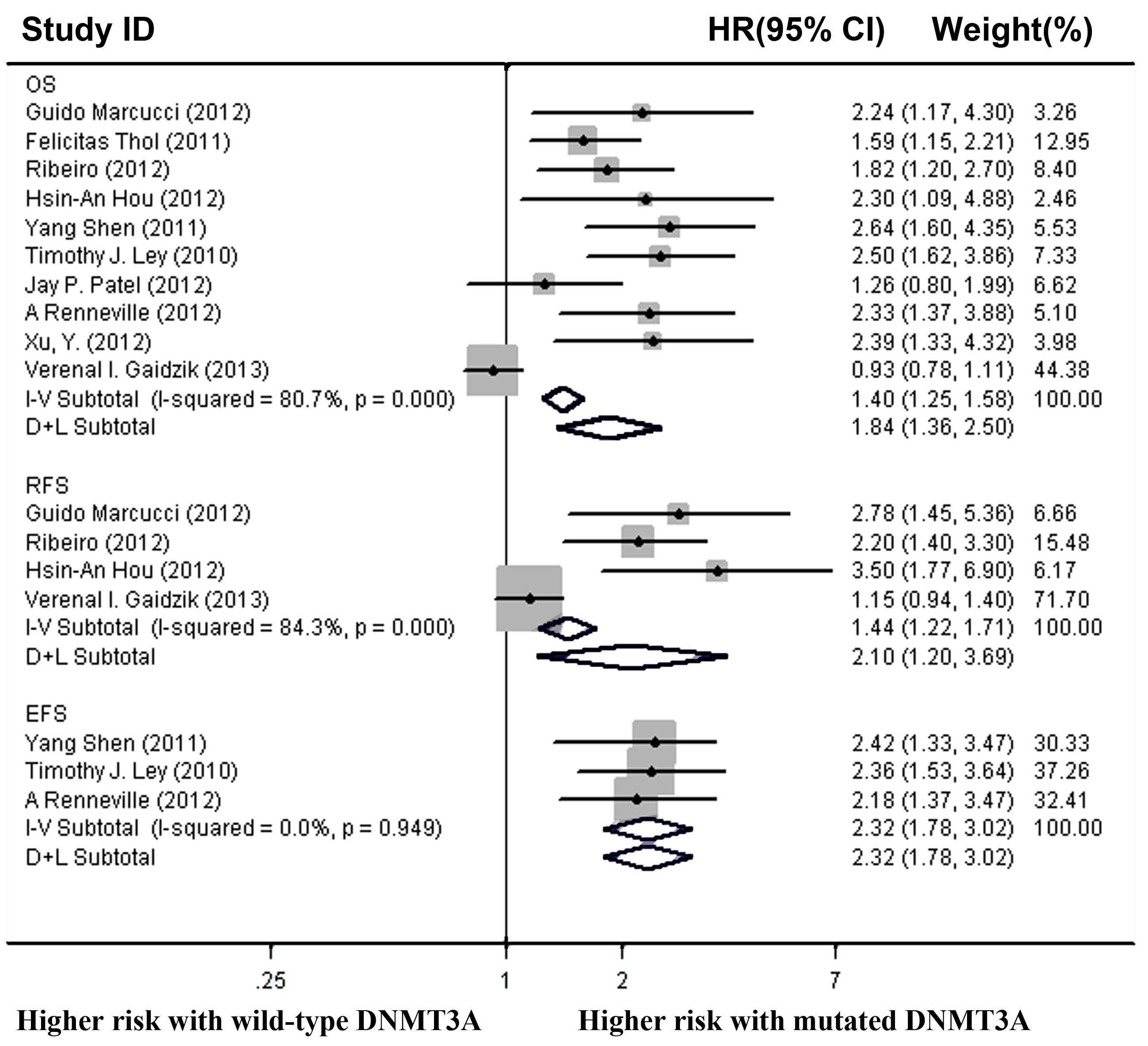

Supplement: Figure S3 — Forest plot of the HRs with 95% CIs for OS, RFS and EFS of patients younger than 60 years of age (mutant DNMT3A versus wild-type DNMT3A). (TIF) [file pone.0093353.s003.tif]

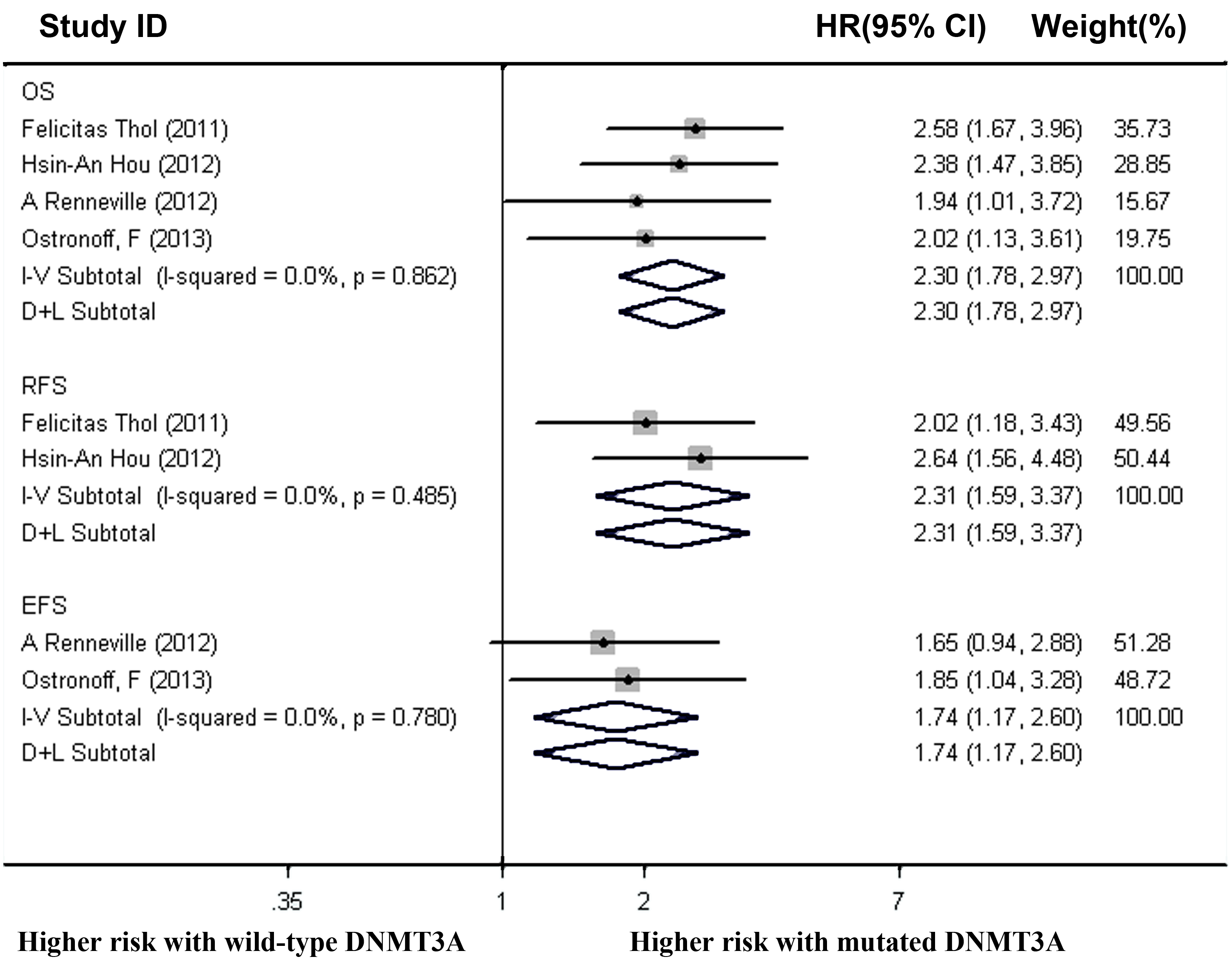

Supplement: Figure S4 — Forest plot of the HRs with 95% CIs for OS, RFS and EFS of patients with unfavorable risk genotype (mutant DNMT3A versus wild-type DNMT3A). (TIF) [file pone.0093353.s004.tif]

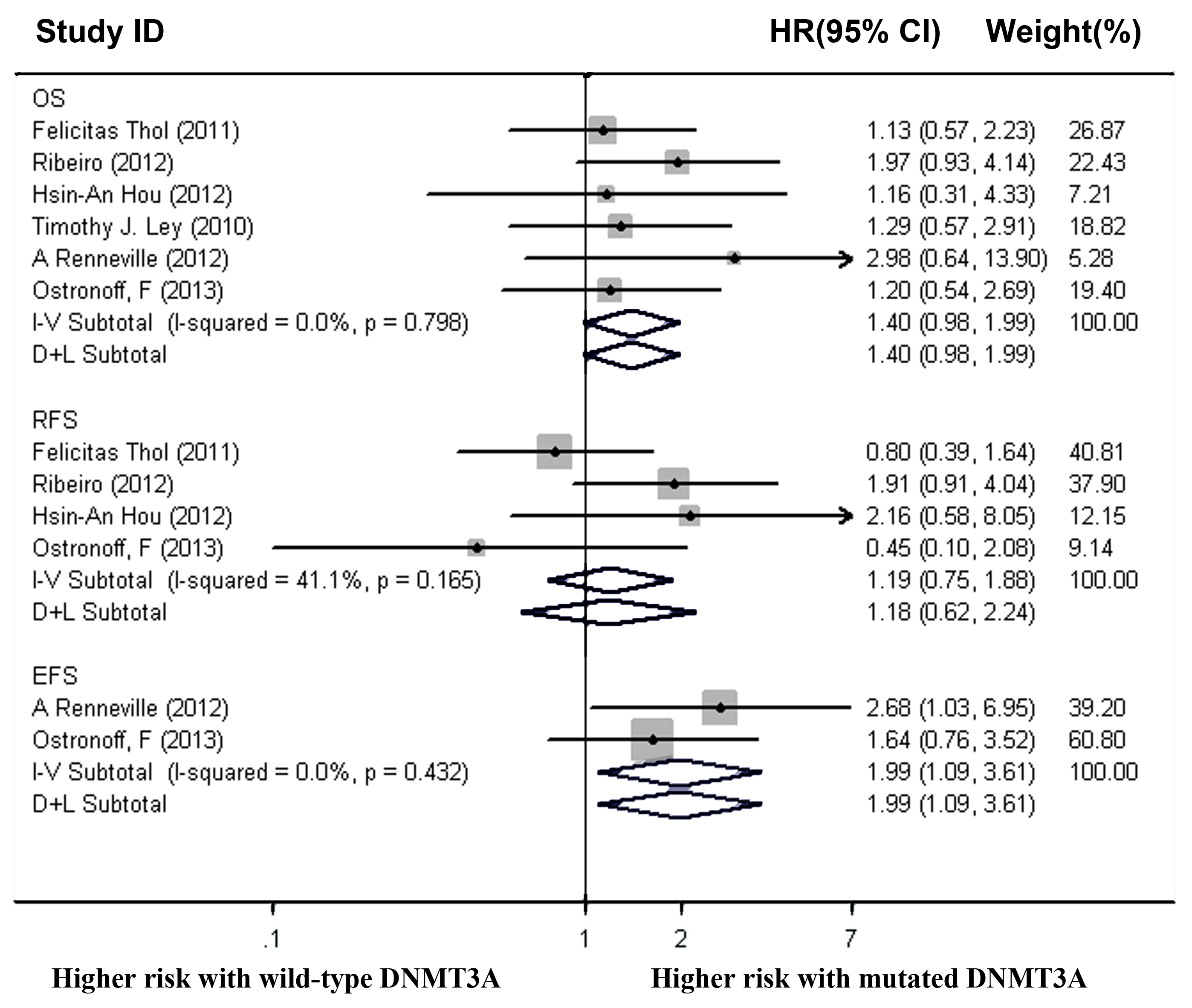

Supplement: Figure S5 — Forest plot of the HRs with 95% CIs for OS, RFS and EFS of patients with favorable risk genotype (mutant DNMT3A versus wild-type DNMT3A). (TIF) [file pone.0093353.s005.tif]

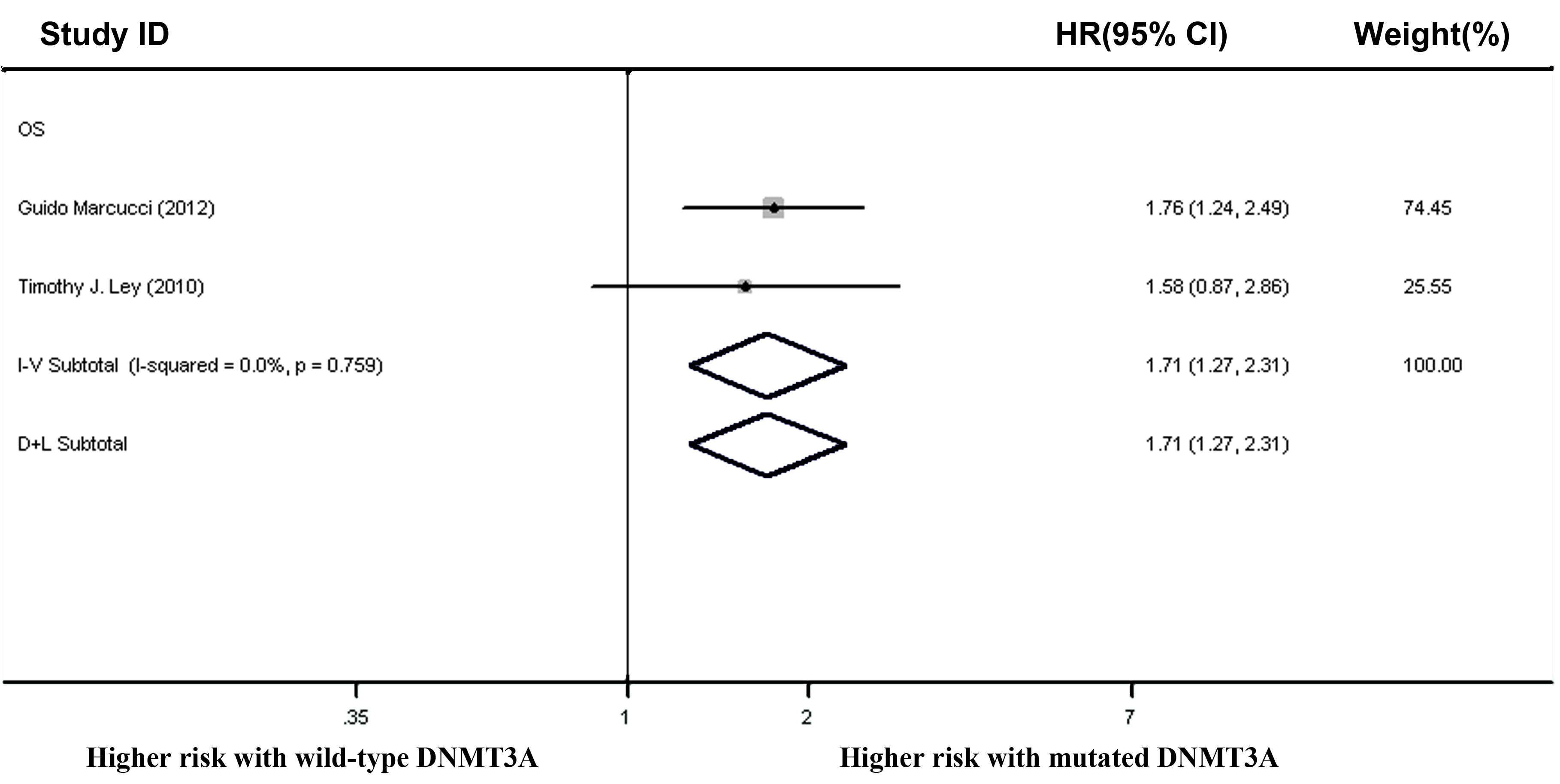

Supplement: Figure S6 — Forest plot of the HR with 95% CI for OS of patients older than 60 years of age (mutant DNMT3A versus wild-type DNMT3A). (TIF) [file pone.0093353.s006.tif]

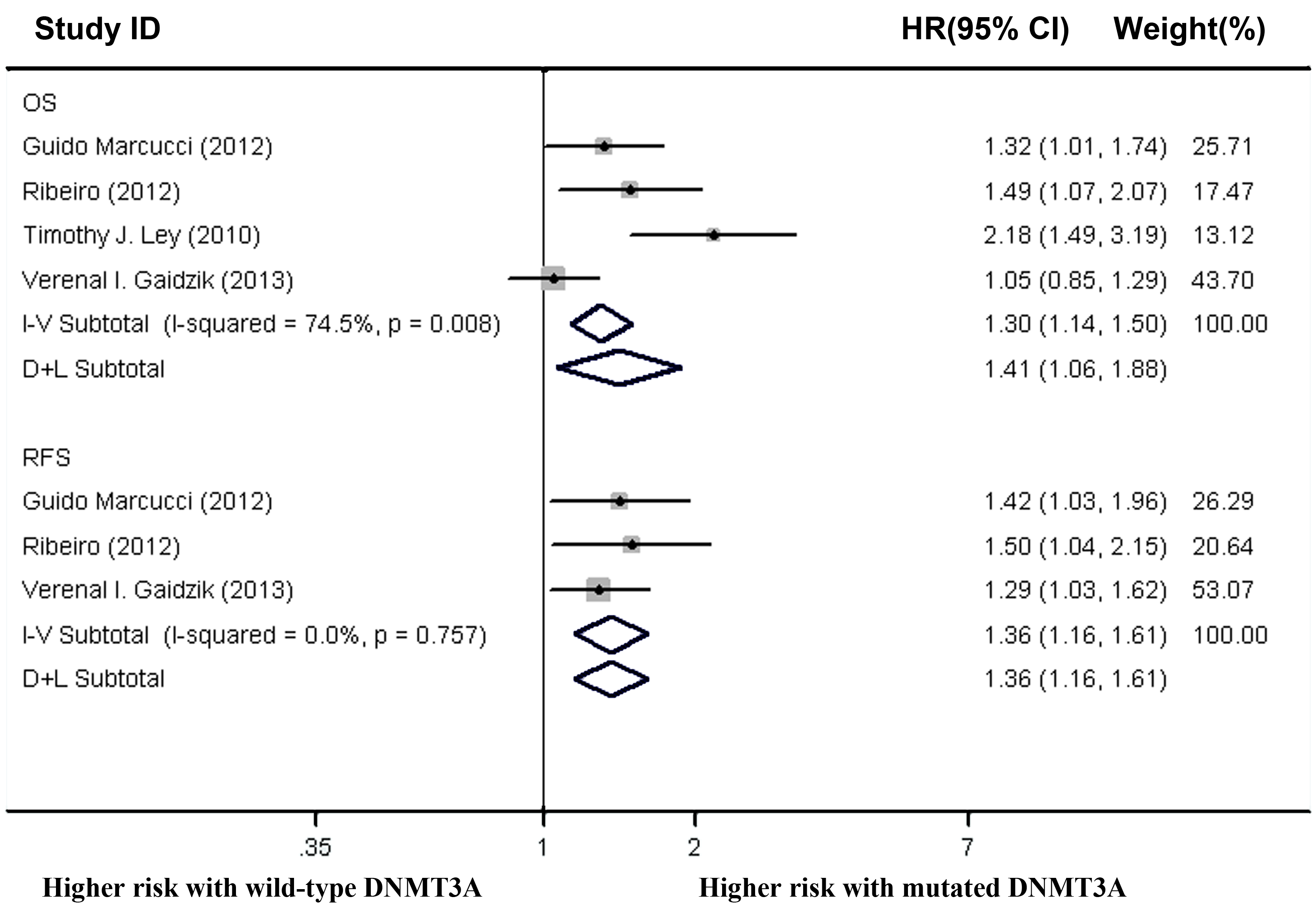

Supplement: Figure S7 — Forest plot of the HRs with 95% CIs for OS and RFS of patients with R882-mutant DNMT3A as compared to patients with wild-type DNMT3A. (TIF) [file pone.0093353.s007.tif]

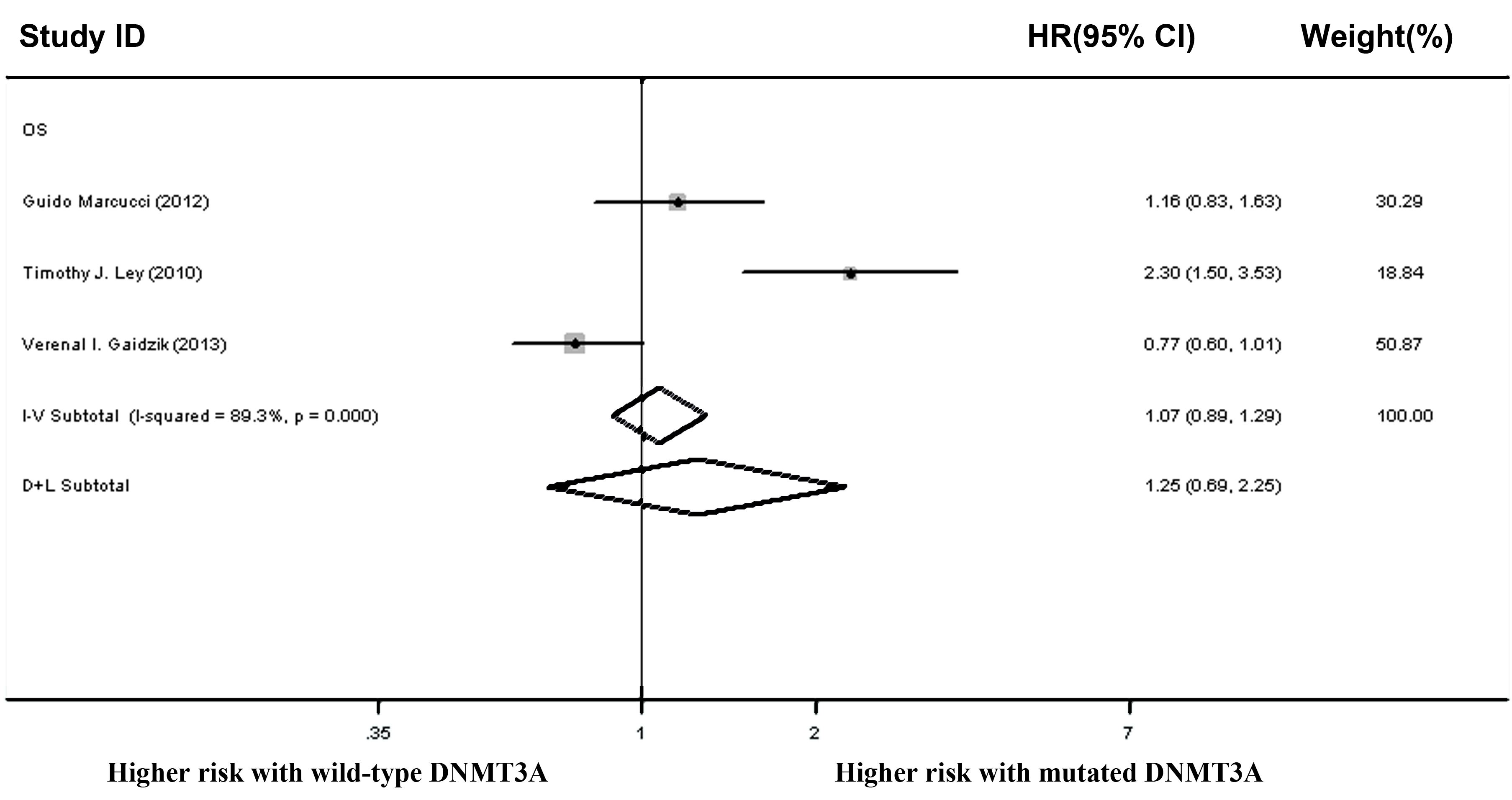

Supplement: Figure S8 — Forest plot of the HR with 95% CI for OS of patients with Non-R882-mutant DNMT3A as compared to patients with wild-type DNMT3A. (TIF) [file pone.0093353.s008.tif]
