# Supplementary material for: Association between DNMT3A Mutations and Prognosis of Adults with De Novo Acute Myeloid Leukemia: A Systematic Review and Meta-Analysis
Source: PLoS One. 2014 Jun 17;9(6):e93353. doi: 10.1371/journal.pone.0093353 (PMC4061003; doi:10.1371/journal.pone.0093353)
Supplement: Table S1 — Clinical and laboratory characteristics of patients with AML with DNMT3A mutations from the 12 included studies. (DOCX) [file pone.0093353.s009.docx]

**Table S1. Clinical and laboratory characteristics of patients with AML with DNMT3A mutations from the 12 included studies.**

|  | | | | | | | | | | |
| --- | --- | --- | --- | --- | --- | --- | --- | --- | --- | --- |
| **First athor** | **ECOG**^◇^  **(N. of P.)** | | **Median percentage of bone marrow blast, %（range）** | | **Median white-cell count, ×10^9^/L (range)** | **Platelet count, ×10^9^/L (range)** | **Cytogenetics** | **Molecular abnormalities** | **NPM1/FLT3 -ITD mutation risk group** | **Treatment protocols** |
| Timothy J. Ley | — | | 69.5 (51.9-87.1) | | 46.4 (5.4-112.0) | — | Normal karyotype, 44; t (15;17), 1; trisomy 8, 5; trisomy 21, 1; trisomy 11,1; complex, 3 | NPM1, 37; FLT3-ITD, 25; IDH1, 13; IDH2, 7 | — | Chemotherapy |
| Yang Shen | — | | 78 (32-97) | | 37.9 (1.1-447.6) | — | — | — | — | Chemotherapy or allo-HSCT^★^ |
| Felicitas Thol | 0 (17); 1 (54);  2 (16);3 (0) | | 78 | | 38 (0.5-328.2) | 71 (9-336) | Normal karyotype, 71 | NPM1, 56; FLT3-ITD, 34; IDH1, 13; IDH2, 9; N-RAS, 8 | a, 53; b, 32; unknown, 2 | Chemotherapy or allo-HSCT |
| Hsin-An Hou | — | 19.03 (0.11-283.21)^◆^ | | | 32.5 (0.65-340.4) | 57 (10-436) | Normal karyotype, 51; del5/del5(q), 1; del7/del7(q), 1; trisomy 8, 4; trisomy 11,1; monosomy, 13; complex,2 | NPM1, 38; FLT3-ITD, 30; FLT3-TKD, 9; IDH1, 4; IDH2, 16; N-RAS, 8; K-RAS, 1; PTPN11, 7; TET2, 6; WT1, 2; CEBPA, 3; MLL-PTD, 6; RUX1, 8; ASXL1, 4 | a, 52; b, 18; unknown, 0 | Chemotherapy or allo-HSCT |
| Jana Markova | — | | — | | 34.5 | — | — | FLT3-ITD, 30 | — | Chemotherapy or allo-HSCT |
| A Renneville | — | | — | | 13 (1-152) | — | Normal karyotype, 36 | NPM1, 24; FLT3-ITD, 7; FLT3-TKD, 3; IDH1, 8; IDH2, 5 | a, 18; b, 18; unknown, 0 | Chemotherapy or allo-HSCT |
| Guido Marcucci | — | | 70 (4-97) | | 43.4 (0.9-434.1) | 66 (4-481) | Normal karyotype, 142 | NPM1, 107; FLT3-ITD, 62; FLT3-TKD, 10; IDH1, 22; IDH2, 24;TET2, 31; WT1, 10; CEBPA , 7; MLL-PTD, 7 | a, 82; b, 60; unknown, 0 | Chemotherapy or auto-HSCT^☆^ |
| Jay P. Patel | — | | — | | — | — | del7/del7(q), 1; trisomy 8, 6; monosomy,1 | NPM1, 57; FLT3-ITD, 53; IDH1, 13; IDH2,6; N-RAS, 10; K-RAS, 2; TET2, 6; WT1, 3; CEBPA, 7; MLL-PTD, 4; KIT, 2; RUX1, 3 | — | Chemotherapy |
| Ana Flávia Tibúrcio Ribeiro | — | | 68 (9-98) | | 52.9 (1.1-278.0) | 64 (10-494) | Normal karyotype, 72; del5/del5(q), 2; del7/del7(q), 3; trisomy 8, 5; 11q23, 1; monosomy, 2; complex, 4 | NPM1, 73; FLT3-ITD, 39; FLT3-TKD, 15; IDH1, 22; IDH2, 13; N-RAS, 8; K-RAS, 3;WT1, 3; CEBPA, 3; KIT, 1 | a, 56; b, 37; unknown, 3 | Chemotherapy or allo-HSCT |
| Xu, Y | — | | — | — | | — | — | — | — |  |
| Ostronoff, F | 0 (7); 1 (20);  2 (8); 3 (2) | | 70 | 37 | | 59 | Normal karyotype, 21 | NPM1, 17; FLT3-ITD, 13; IDH2, 6 | a, 14; b, 12; unknown, 9 | Chemotherapy |
| Verena I. Gaidzik | — | | 80 (2-100) | 24.5 (0.2-532) | | 70 (5-916) | Normal karyotype, 268; inv(3)/t(3;3), 4; trisomy 8, 17; 11q23, 3; complex,13 | NPM1, 234; FLT3-ITD, 126; FLT3-TKD, 28; IDH1, 37; IDH2, 59; CEBPA, 18 | — | Chemotherapy or allo-HSCT |

◇, ECOG performance status indicates Eastern Cooperative Oncology Group; ◆, absolute count, ×10^9^/L;

a, is defined as either wild-type NPM1 regardless of the status of FLT3-ITD or positive FLT3-ITD regardless of the status of NPM1;

b, is defined as mutant NPM1 and the absence of FLT3-ITD; —, indicates there is no related data presented;

★^, allogeneic hematopoietic stem cell transplantation;^

☆^, autologous hematopoietic stem cell transplantation.^
