# Supplementary material for: Association between DNMT3A Mutations and Prognosis of Adults with De Novo Acute Myeloid Leukemia: A Systematic Review and Meta-Analysis
Source: PLoS One. 2014 Jun 17;9(6):e93353. doi: 10.1371/journal.pone.0093353 (PMC4061003; doi:10.1371/journal.pone.0093353)
Supplement: Table S2 — Clinical and laboratory characteristics of patients with AML without DNMT3A mutations from the 12 included studies. (DOCX) [file pone.0093353.s010.docx]

**Table S2. Clinical and laboratory characteristics of patients with AML without DNMT3A mutations from the 12 included studies.**

|  | | | | | | | | | | | |
| --- | --- | --- | --- | --- | --- | --- | --- | --- | --- | --- | --- |
| **First athor** | **ECOG**^◇^  **(N. of P.)** | **Median percentage of bone marrow blast, % （range）** | | **Median white-cell count, ×10^9^/L (range)** | | **Platelet count, ×10^9^/L (range)** | **Cytogenetics** | **Molecular abnormalities** | | **NPM1/FLT3 -ITD mutation risk group** | **Treatment protocols** |
| Timothy J. Ley | — | | 69.4 (50.8-88.0) | 11.8 (26.5-105.1) | | — | Normal karyotype, 76; t (15;17), 45; t (8;21), 12; inv (16)/t (16;16), 20; del5/del5(q), 1; del7/del7(q), 7; inv(3)/t(3;3), 2; trisomy 8, 6; tisomy 21, 5; trisomy 11,1; trisomy13, 3; complex, 20 | | NPM1, 27; FLT3-ITD, 48; IDH1, 12; IDH2, 13 | — | Chemotherapy |
| Yang Shen | — | | 65.0 (22.5-97.0) | 37.9 (1.1-447.6) | | — | — | | — | — | Chemotherapy or allo-HSCT^★^ |
| Felicitas Thol | 0 (101);  1 (247); 2 (50) | | 70 | 17.3 (0.5-284) | 44 (3-624) | | Normal karyotype, 190 | | NPM1, 107; FLT3-ITD, 95; IDH1, 22; IDH2, 26; N-RAS, 49 | a, 335; b, 62; unknown, 5 | Chemotherapy or allo-HSCT |
| Hsin-An Hou | — | | 6.26 (0-456.73)^◆^ | 15.9 (0.12-627.8) | | 39 (2-802) | Normal karyotype, 172; t(15;17), 38; t(8;21), 42; inv(16)/t(16;16), 19; del5/del5(q), 1; del7/del7(q), 9; trisomy 8, 23; tisomy 21, 9; trisomy 11, 2; trisomy 13, 1; 11q23,16; monosomy, 195; complex ,20 | | NPM1, 66; FLT3-ITD, 83; FLT3-TKD, 29; IDH1, 23; IDH2, 39; N-RAS, 53; K-RAS, 15; PTPN11, 11; TET2, 59; WT1, 31; CEBPA , 63; MLL-PTD, 21; KIT , 15; RUX1, 54; ASXL1, 46; JAK2, 3 | — | Chemotherapy or allo-HSCT |
| Jana Markova | — | | — | 15 | | — | — | | FLT3-ITD,42 | — | Chemotherapy or allo-HSCT |
| A Renneville | — | | — | 11.0 (0.7-250) | | — | Normal karyotype, 87 | | NPM1, 28; FLT3-ITD, 18; FLT3-TKD,2; IDH1, 9; IDH2, 14;WT1, 7; CEBPA , 12; | a, 16; b, 26 | Chemotherapy or allo-HSCT |
| Guido Marcucci | — | | 66 (7-96) | 22.4 (0.9-450) | | 61 (7-850) | Normal karyotype, 273 | | NPM1, 146; FLT3-ITD, 85; FLT3-TKD, 21; IDH1, 26; IDH2, 51; TET2, 67; WT1, 28; CEBPA , 58; MLL-PTD, 15 | a, 133; b, 140 | Chemotherapy or auto-HSCT^☆^ |
| Jay P. Patel | — | | — | — | | — | — | | — | — | Chemotherapy |
| Ana Flávia Tibúrcio Ribeiro | — | | 68 (0-98) | 52.9 (1.1-278.0) | | 64 (10-494) | Normal karyotype,122; t(8;21), 35; inv(16)/t(16;16), 34; del5/del5(q), 13; del7/del7(q), 24; inv(3)/t(3;3), 5; trisomy 8, 27; tisomy 21, 7; 11q23,25; monosomy, 22; complex, 34 | | NPM1, 60; FLT3-ITD, 77; FLT3-TKD, 26; IDH1, 11; IDH2, 23; N-RAS, 34; K-RAS, 1;WT1, 21; CEBPA, 28; KIT, 22 | — | Chemotherapy or allo-HSCT |
| Xu, Y | — | | — | — | | — | — | | — | — |  |
| Ostronoff, F | 0 (34); 1 (74);  2 (23); 3 (20) | | 71 | 28 | | 60 | Normal karyotype, 45 | | NPM1, 36; FLT3-ITD, 34; IDH1, 5; IDH2, 34; CEBPA , 3 | a, 110; b, 18; unknown, 26 | Chemotherapy |
| Verena I. Gaidzik | — | | 73 (2-100) | 10.3 (0.2-372) | | 49 (2-993) | Normal karyotype, 526; inv(3)/t(3;3), 26; trisomy 8, 6; 11q23, 27; complex,162 | | NPM1, 241; FLT3-ITD, 261; FLT3-TKD, 97; IDH1, 62; IDH2, 89; CEBPA, 105 | — | Chemotherapy or allo-HSCT |

◇, ECOG performance status indicates Eastern Cooperative Oncology Group; ◆, absolute count, ×10^9^/L;

a, is defined as either wild-type NPM1 regardless of the status of FLT3-ITD or positive FLT3-ITD regardless of the status of NPM1;

b, is defined as mutant NPM1 and the absence of FLT3-ITD; —, indicates there is no related data presented;

★^, allogeneic hematopoietic stem cell transplantation;^

☆^, autologous hematopoietic stem cell transplantation.^
